# A Small Number of Low-abundance Bacteria Dominate Plant Species-specific Responses during Rhizosphere Colonization

**DOI:** 10.3389/fmicb.2017.00975

**Published:** 2017-05-29

**Authors:** Wayne Dawson, Jens Hör, Markus Egert, Mark van Kleunen, Michael Pester

**Affiliations:** ^1^Department of Biosciences, Durham UniversityDurham, United Kingdom; ^2^Department of Biology, University of KonstanzKonstanz, Germany; ^3^Faculty of Medical and Life Sciences, Institute of Precision Medicine, Furtwangen UniversityVillingen-Schwenningen, Germany

**Keywords:** microbiome, next-generation amplicon sequencing, rhizosphere, 16S rRNA gene, rare biosphere

## Abstract

Plant growth can be affected by soil bacteria. In turn, plants are known to influence soil bacteria through rhizodeposits and changes in abiotic conditions. We aimed to quantify the phylotype richness and relative abundance of rhizosphere bacteria that are actually influenced in a plant species-specific manner and to determine the role of the disproportionately large diversity of low-abundance bacteria belonging to the rare biosphere (<0.1 relative abundance) in this process. In addition, we aimed to determine whether plant phylogeny has an influence on the plant species-specific rhizosphere bacterial community. For this purpose, 19 herbaceous plant species from five different plant orders were grown in a common soil substrate. Bacterial communities in the initial soil substrate and the established rhizosphere soils were compared by 16S rRNA gene amplicon sequencing. Only a small number of bacterial operational taxonomic units (OTUs, 97% sequence identity) responded either positively (ca. 1%) or negatively (ca. 1%) to a specific plant species. On average, 91% of plant-specific positive response OTUs comprised bacteria belonging to the rare biosphere, highlighting that low-abundance populations are metabolically active in the rhizosphere. In addition, low-abundance OTUs were in terms of their summed relative abundance major drivers of the bacterial phyla composition across the rhizosphere of all tested plant species. However, no effect of plant phylogeny could be observed on the established rhizosphere bacterial communities, neither when considering differences in the overall established rhizosphere communities nor when considering plant species-specific responders only. Our study provides a quantitative assessment of the effect of plants on their rhizosphere bacteria across multiple plant orders. Plant species-specific effects on soil bacterial communities involved only 18–111 bacterial OTUs out of several 1000s; this minority may potentially impact plant growth in plant–bacteria interactions.

## Introduction

The performance and growth of plants is tightly connected to chemical, physical, and biological properties of the inhabited soil. In turn, plants directly influence abiotic and biotic soil properties (e.g., van der Putten et al., 2013). For example, plants invest 11–40% of their photosynthetically fixed carbon and 10–16% of total plant nitrogen into rhizodeposits that modulate the microbiota in their close root vicinity (Berendsen et al., 2012; Bulgarelli et al., 2013). Such rhizodeposits include inorganic ions, organic acids, sugars, purines, and nucleosides but also secondary metabolites like vitamins, phytosiderophores, antibiotics, and quorum-sensing mediating compounds (Berendsen et al., 2012; Bulgarelli et al., 2013). In addition, plant roots modulate their microbiota by influencing the soil pH, soil structure and oxygen availability of their surrounding soil (Marschner et al., 1986; Dennis et al., 2010).

Through these mechanisms, several plant species including *Arabidopsis thaliana* (thale cress), *Zea mays* (maize), *Solanum tuberosum* (potato), and *Hordeum vulgare* (barley) (Weinert et al., 2011; Bulgarelli et al., 2012, 2015; Lundberg et al., 2012; Peiffer et al., 2013) have been shown to modify the composition and activity of the soil microbiota in close proximity to their roots (rhizosphere) and directly on or inside their roots (Bulgarelli et al., 2013; Philippot et al., 2013). For example, symbionts such as the N_2_-fixing *Bradyrhizobium japonicum* and pathogens such as *Phytophthora sojae* are attracted by specific plant flavonoids (Philippot et al., 2013). In more general terms, a step-wise selection model has been proposed for root microbiota differentiation (Bulgarelli et al., 2013; Reinhold-Hurek et al., 2015). In the initial soil, edaphic factors (e.g., soil pH and structure) determine the composition of the soil microbial community. The first selection step takes place in the rhizosphere, the thin soil layer directly surrounding the roots, where rhizodeposits and root cell wall features promote the growth of mainly organotrophic microorganisms and thereby initiate a shift in the soil microbiome. In a second and third step, a host genotype-dependent selection takes place close to and within the root corpus, respectively, and thereby fine-tunes community profiles thriving on the rhizoplane (i.e., the root surface) and within plant roots (Bulgarelli et al., 2013; Reinhold-Hurek et al., 2015).

The bacterial domain harbors an exceptionally high species richness among soil microorganisms (Curtis et al., 2002; Schloss et al., 2016) as compared to archaea (Schloss et al., 2016) and fungi (Bridge and Spooner, 2001; Fierer et al., 2007). The question whether plant species differentiate their root bacteria in a species-specific manner has been addressed in various studies over the past decades. Here, cultivation efforts as well as molecular fingerprinting techniques such as DGGE (denaturing gradient gel electrophoresis), T-RFLP (terminal restriction fragment length polymorphism) or PLFA (phospholipid-derived fatty acids) analysis revealed that different plant species can indeed select for specific rhizosphere bacteria (e.g., Miethling et al., 2000; Smalla et al., 2001; Garbeva et al., 2008; Berg and Smalla, 2009; Bezemer et al., 2010; Pendergast et al., 2013). However, only few studies extended this knowledge so far to a more complete census of the plant species-specific rhizosphere bacterial community using modern next-generation amplicon sequencing approaches that target the phylogenetic breadth of most bacteria at sufficient sampling depth (Rodríguez-Echeverría et al., 2013; Rosenzweig et al., 2013; Hortal et al., 2015). Furthermore, studies on the role of low-abundance bacterial populations in shaping the plant species-specific root bacteriome are still scarce (Dohrmann et al., 2013; Nuccio et al., 2016; Saleem et al., 2016; Shi et al., 2016).

Next-generation amplicon sequencing can generate several millions of sequences over several 100 multiplexed samples (Caporaso et al., 2011, 2012). This approach was a breakthrough to address microbial ecology questions in a (semi-)quantitative and well-replicated manner, especially for highly complex bacterial communities such as those inhabiting soil environments. Soils are typically inhabited by several 1000s of bacterial species, which exhibit a continuous but highly skewed rank abundance (e.g., Lauber et al., 2009; Nemergut et al., 2010; Hausmann et al., 2016; Wörner et al., 2016). A minor proportion of these taxa are very or moderately abundant (≥1 and ≥0.1% relative abundance, respectively) and are typically thought to be responsible for major ecosystem functions. The remaining major part of these species have an individual relative abundance of <0.1% and are defined as the rare biosphere (Sogin et al., 2006), which is typically thought of as a ‘seed’ bank for the recruitment of dormant cells to become metabolically active and numerically dominant after environmental conditions change (Pedrós-Alió, 2012; Lynch and Neufeld, 2015). However, not all low-abundance bacteria are metabolically inactive and there are a few documented cases where they even can fulfill important ecosystem functions, e.g., as N_2_-fixing bacteria in the ocean (Großkopf et al., 2012) or as sulfate-reducing bacteria in peatland soils (Pester et al., 2010; Hausmann et al., 2016). For soil environments, additional ecosystem functions have been demonstrated for bacterial populations of low relative abundance (reviewed in Jousset et al., 2017). For example, removal of low-abundance species from bulk soil using dilution-to-extinction had a positive effect on the establishment of newly introduced species, suggesting that low-abundance species pre-occupy ecological niches and thus slow down invasion by incoming species (van Elsas et al., 2012; Vivant et al., 2013; Mallon et al., 2015). Soil microorganisms of low relative abundance were also shown to play a role in community-wide species interactions, e.g., by being involved in the production of antifungal compounds that protect plants from pathogens (Hol et al., 2015). However, how taxa within this rare biosphere respond to growth of different plant species is not well-understood.

To assess how the composition of the rhizosphere bacterial community is shaped by different plant species, we used 19 herbaceous plant species grown independently in a common soil substrate. Our objectives were to (i) quantify how many species-level bacterial taxa respond in a plant-species specific manner, (ii) address the role of the rare biosphere in shaping the rhizosphere bacterial community, and (iii) determine whether plant phylogeny has an influence on the rhizosphere bacterial community.

## Materials and Methods

### Plant Mesocosms

We used 19 herbaceous, mostly perennial grassland species (**Table 1**) that had been previously analyzed in a plant-soil feedback experiment for their relationship between specific growth rates and biotic soil effects by Lemmermeyer et al. (2015). Seeds were collected in 2012 in Switzerland and southern Germany (both in the vicinity of Konstanz) from 3 to 10 mother plants per species originating from one population. Equal numbers of seeds per mother plant were then pooled to get one bulk sample per species.

**Table 1:** Overview on the taxonomy and growth form of the 19 studied grassland plant species.

Species	Family	Order	Growth form	Mean aboveground biomass (g)	Nr. of soil replicates	
					
					Initial	Rhizosphere
*Agrostis capillaris*	Poaceae	Poales	Perennial grass	5.00 (1.45)	1	5
*Brachypodium sylvaticum*	Poaceae	Poales	Perennial grass	1.65 (1.06)	4	5
*Dactylis glomerata*	Poaceae	Poales	Perennial grass	7.07 (2.60)	4	5
*Deschampsia cespitosa*	Poaceae	Poales	Perennial grass	2.19 (0.77)	4	4
*Phleum pratense*	Poaceae	Poales	Perennial grass	4.71 (1.69)	5	4
*Centaurea jacea*	Asteraceae	Asterales	Perennial forb	4.18 (0.51)	5	5
*Cirsium oleraceum*	Asteraceae	Asterales	Perennial forb	5.06 (1.67)	5	4
*Jacobaea vulgaris*	Asteraceae	Asterales	Perennial forb	1.68 (1.57)	4	5
*Pulicaria dysenterica*	Asteraceae	Asterales	Perennial forb	5.96 (1.48)	4	5
*Taraxacum officinale*	Asteraceae	Asterales	Perennial forb	5.08 (1.05)	5	5
*Rumex maritimus*	Polygonaceae	Caryophyllales	Perennial forb	7.25 (1.83)	5	3
*Rumex obtusifolius*	Polygonaceae	Caryophyllales	Perennial forb	4.27 (1.80)	5	5
*Silena alba*	Caryophyllaceae	Caryophyllales	Perennial forb	6.32 (0.81)	5	5
*Silene vulgaris*	Caryophyllaceae	Caryophyllales	Perennial forb	3.72 (1.83)	5	5
*Plantago lanceolata*	Plantaginaceae	Lamiales	Perennial forb	9.29 (1.28)	5	5
*Plantago major*	Plantaginaceae	Lamiales	Perennial forb	3.52 (0.73)	5	3
*Verbascum thapsus*	Scrophulariaceae	Lamiales	Biennial forb	4.94 (2.58)	5	5
*Salvia pratensis*	Lamiaceae	Lamiales	Perennial forb	4.92 (1.59)	5	5
*Lotus corniculatus*	Fabaceae	Fabales	Perennial forb	9.32 (2.66)	5	5


*The mean (±1 standard deviation) of aboveground biomass of plants after a growth period of 14 weeks and the final number of sequenced biological replicates from initial soil substrate and rhizosphere soils are given as well.*

In May 2013, 100 seeds were sown in five 4.5-L pots per species, giving five independent replicates per plant species. The substrate in the pots consisted of 4 L of a mixture of topsoil (‘Rasenerde,’ Ökohum GmbH, Herbertingen, Germany), vermiculite (type ‘palabora,’ 0–1 mm, Deutsche Vermiculite Dämmstoff GmbH, Sprockhövel, Germany) and washed sand (Type 22200, 0.3–0.8 mm, Emil Steidle GmbH & Co. KG, Sigmaringen, Germany) (ratio of 1:1:1 by volume). With the aim of providing the plants with an inoculum of a soil microbial community native to the plant species, we mixed in 200 ml of homogenized soil collected from seven grassland areas in the vicinity of Konstanz (coordinates: 47°41′26″ N, 9°11′27″ E; 47°41′18″ N, 9°11′28″ E; 47°41′16″ N, 9°11′34″ E; 47°41′13″ N, 9°11′18″ E; 47°41′10″ N, 9°11′26″ E; 47°40′59″ N, 9°11′25″ E; 47°40′39″ N, 9°11′46″ E). The soils in this region are brown earths, and predominantly loamy in texture (Hartwich et al., 2013). Here, soil of approximately 0–10 cm depth was obtained from 12 to 20 points located at regular intervals (5 m) along line transects in each grassland area. This yielded a total of ∼40 L of soil, which was bulked, sieved (using a 5-mm mesh to remove plant roots and stones) and mixed thoroughly before mixing it with the rest of the substrate in the pots. This gave a total of 95 pots, all of which initially received the same homogenized soil substrate mixture. Before sowing seeds of the 19 species into five replicate pots each, every pot was assigned to one of the species as a replicate, and a sample of the prepared soil substrate was taken from the center of each pot to a depth of 10 cm using a sterile 2-cm diameter soil corer. Each of these initial soil substrate samples was stored individually at -80°C until DNA extraction (see below). The hole was then filled in with surrounding soil substrate, and seeds were sown.

After germination, seedlings were thinned to a constant density of five plants per pot. Resulting plants were then grown outside in the botanical garden of the University of Konstanz initially for 10 weeks. Natural rainfall was supplemented with watering during dry periods. For an additional 4 weeks, pots were placed in a glasshouse to promote further growth until 17–18 September 2013. Throughout this period, newly germinating seedlings of target and non-target plants were constantly removed. At the end of the experiment, the aboveground biomass per pot was harvested, and pot contents were removed immediately. Aboveground biomass was dried at 70°C for 72 h and later weighed to an accuracy of 1 mg. We carefully separated the roots from the bulk of the soil substrate, then gently shook roots to dislodge attached soil (rhizosphere soil), and collected the rhizosphere-soil samples (one per individual pot, 10–20 ml in volume) into sterile 50-mL conical centrifuge tubes (Carl Roth GmbH, Karlsruhe, Germany). These were stored at -80°C until required for DNA extraction.

### DNA Extraction and Amplicon Sequencing

We extracted DNA from 0.25 g of each initial soil substrate and rhizosphere soil sample using the PowerSoil DNA Isolation Kit (MOBIO Laboratories, Inc., Carlsbad, CA, United States), following the manufacturer’s instructions. To characterize the bacterial community of each sample, 16S rRNA genes were amplified according to the manual of the NEXTflex^TM^ 16S V1–V3 amplicon sequencing kit (Bioo Scientific, Austin, TX, United States), which included the preparation of a multiplexed amplicon library for paired-end sequencing on the Illumina^®^ MiSeq platform. In brief, 25 ng of total extracted DNA were used as template for amplifying the V1–V3 region of bacterial 16S rRNA genes using the universal primers 8f (5′-AGAGTTTGATCCTGGCTCAG-3′) and 536r (5′-GTATTACCGCGGCTGCTGG-3′) that were elongated by oligonucleotide adaptors for subsequent PCR barcoding. This PCR consisted of an initial denaturation at 98°C for 4 min followed by 10 cycles of denaturation at 98°C for 30 s, annealing at 60°C for 30 s, and elongation at 72°C for 30 s with a final elongation for 4 min at 72°C. PCR products were purified using Agencourt AMPure XP Magnetic beads (Beckman Coulter, Indianapolis, IN, United States) and used as template in a subsequent PCR with 14 cycles to introduce barcodes and Illumina sequencing adaptors according to the manufacturer’s instructions. PCR conditions were the same as outlined for the first PCR. Amplicon sequencing was performed at Furtwangen University on the Illumina MiSeq platform using paired-end sequencing (2 × 300 bp) based on the MiSeq Reagents kit v3.

Amplicon reads were processed in mothur v.1.34.4 (Schloss et al., 2009) including quality control, removal of unique singletons, *de novo* chimera filtering using UCHIME (Edgar et al., 2011), *de novo* clustering of operational taxonomic units (OTUs) at 97% sequence identity (approximate species level), and removal of OTUs with fewer than five reads to discriminate against sequencing artifacts and sample bleeding (Mitra et al., 2015). This resulted in 6.1 million high-quality paired-end reads forming 18838 OTUs. The library sizes had on average 34552 paired-end reads per sample, with 90% of all samples having library sizes between 22514 and 53707 paired-end reads (5 and 95% quantiles, respectively). Good’s coverage (Good, 1953) of processed reads was on average 95%, with 90% of all samples having a coverage between 94 and 98% (5 and 95% quantiles, respectively). Individual sequences were classified with the Bayesian classifier and the RDP 16S rRNA training set 14 (Cole et al., 2014) as implemented in mothur using a k-mer size of 8 and a confidence threshold of 80. Illumina sequences were deposited at the Sequence Read Archive at NCBI under accession number SRP081003.

### Quantitative PCR

A quantitative PCR (qPCR) assay targeting most bacteria and archaea was performed following the procedure described by Hausmann et al. (2016) using the universal primers 1389F (5′-TGYACACACCGCCCGT-3′) and modified 1492R (5′-NTACCTTGTTACGACT-3′). qPCRs were performed in 20-μl reactions on a 7500 Fast Real-Time PCR System (Applied Biosystems, Foster City, CA, United States) using the GoTaq qPCR Master Mix (Promega, Madison, WI, United States), bovine serum albumine (10 ng μl^-1^), 0.8–6.3 ng of template DNA and the following primer end-concentrations: 1389F (0.75 μM) and 1492R (1.00 μM). Thermal cycling was carried out by an initial denaturation step at 95°C for 5 min, followed by 40 cycles of denaturation at 95°C for 30 s, annealing at 50°C for 30 s, and elongation at 72°C for 60 s. A standard curve with a purified 16S rRNA gene-PCR product of a *Nitrosomonas urea*-clone was generated from 1.84 × 10^2^ to 1.84 × 10^9^ template copies per assay (*R*^2^ = 0.993–0.998) in a background of 5 ng of purified λ-phage DNA *c*I857 *Sam7* (Promega). PCR efficiencies of the assays ranged from 83 to 91%. qPCR products were regularly checked for specificity by (i) comparative melting-curve analyses of the sample-derived PCR products and the respective reference-derived PCR products and (ii) agarose gel electrophoresis. Partial inhibition of the qPCR was not evident when testing the serial dilution of DNA extracted from a representative soil sample. To estimate the size of overall bacterial and archaeal populations inhabiting the analyzed soils, the retrieved 16S rRNA gene copies per ng total DNA were normalized against the amount of DNA extracted from 250 mg of soil. We assumed a DNA extraction efficiency of 100%.

### Statistical Analysis

All analyses were performed using the program R, versions 3.1.1 and 3.3.0 (R Core Team, 2015). Only OTUs that were detected by at least three reads in three different soil replicates were used to further discriminate against sequencing artifacts and to enable meaningful statistical analysis. Alpha diversity metrics were calculated in the R package phyloseq 1.8.2 (McMurdie and Holmes, 2013) based on uniform library sizes of 16417 reads as obtained by rarefaction back-sampling. Differences in bacterial community composition were visualized using non-metric multidimensional scaling (NMDS) plots made in phyloseq using Bray–Curtis dissimilarities and relative abundance data normalized by the number of reads per soil replicate. Variation in bacterial community composition explained by plant-species identity, sampling time (initial soil substrate or rhizosphere soil), and the interaction between them was tested for significance using the Bray–Curtis dissimilarities and permutational analysis of variance (PERMANOVA, function ‘adonis’ in the R package ‘vegan’ version 2.4-1; Oksanen et al., 2013). The level of dissimilarity among sample communities was compared to a distribution of dissimilarities obtained from 9999 randomisations. *Agrostis capillaris* was excluded from this analysis due to an insufficient number of soil replicates from the initial soil substrate. To assess whether greater plant growth and biomass accumulation resulted in greater shifts in rhizosphere bacterial community composition, we performed another PERMANOVA using Bray–Curtis dissimilarities as above, but only for the rhizosphere soils, and with aboveground biomass as the explanatory variable and species as a random effect.

Differences in variability of bacterial community compositions among samples within groups (initial soil substrate or rhizosphere soils of each species) were assessed using the ‘betadisper’ function in ‘vegan.’ This was done by measuring the distance in multivariate space between each group’s sample community compositions and the group’s centroid. The amount of heterogeneity among plant species in their bacterial community dispersion as well as between initial soil substrate and rhizosphere soils was compared to that obtained from 9999 randomisations using the function ‘permutest.’ A significant *p*-value (*p* < 0.05) would indicate that the within-group variation of bacterial community composition differs significantly among groups.

To test for differential abundance of OTUs in the initial soil substrate versus rhizosphere soils, we used the general linear model approach in combination with quasi-likelihood shrunken dispersion estimates of the R package edgeR (Lund et al., 2012). This analysis was based on RLE (relative log expression) normalized abundance data (Anders and Huber, 2010). OTUs were considered to respond significantly in terms of their relative abundance change if their false detection rate-corrected *p*-value was below 0.01 (i.e., positive and negative responses increased and decreased significantly in relative abundance, respectively). These tests effectively represent paired tests comparing OTU abundances in initial soil substrate versus rhizosphere soils from the same pots. We repeated the PERMANOVA described above for the subsets of bacterial OTUs that responded either commonly to all plant species or specifically to one plant species only using the rhizosphere soils. We did this to assess how much variation in these subsets was explained by plant identity.

Finally, in order to assess whether similarity in rhizosphere bacterial community composition among samples was correlated with phylogenetic similarity among plants, we applied a Mantel test (with a Spearman’s correlation coefficient) on the phylogenetic correlation matrix of the plants, and a Bray–Curtis dissimilarity matrix of the rhizosphere bacterial community. The phylogeny was created by pruning the aged phylogeny of European plant species (Durka and Michalski, 2012), and by adding extra branches representing the replicate samples within each species. Two Mantel tests were performed: one with a rhizosphere bacterial community dissimilarity matrix based on all OTUs, and a second based only on the plant species-specific responding OTUs. This tests involved 9999 random permutations of rows in the dissimilarity matrix, producing a null distribution of correlations to assess observed correlation significance. The phylogeny was built using the package ‘ape’ version 3.5 (Paradis et al., 2004), and the Mantel test was performed using the function ‘mantel’ in the package ‘vegan’ version 2.4-1 (Oksanen et al., 2013).

## Results

### Plant Species Identity Is a Minor Driver of Bacterial Rhizosphere Communities

We used next-generation amplicon sequencing of bacterial 16S rRNA genes (V1–V3 region) to assess the influence of 19 different herbaceous plant species on their own rhizosphere bacterial community. This was contrasted with the initial soil substrate that was used to grow these plants. To maximize detection of plant species influence and to provide equal starting conditions to all plants, this initial soil substrate was made of two components. The major component was a mixture of commercial topsoil, vermiculite and washed sand (ratio 1:1:1), which was foreign to all plant species and used as a background substrate. The minor component was a small inoculum of homogenized soil native to the investigated plant species.

Bacterial alpha diversity in the rhizosphere of plants was clearly different from that in the initial soil substrate irrespective of the plant species (**Supplementary Figure S1**). When rarefied to an equal sampling depth of 16417 reads per replicate, soils harbored on average 2181 ± 246 (mean ± SD) Chao1-estimated OTUs (97% sequence identity) in the initial soil substrate, which slightly but significantly increased to an average of 2727 ± 286 OTUs in rhizosphere soils (ANOVA: *F*_1,136_ = 194.97, *p* < 0.001). Incubation time explained 58% of the observed variation in OTU richness, while 13% of the variation could be attributed to individual plant species (ANOVA: *F*_17,136_ = 2.41, *p* < 0.01). There was no significant interaction between incubation *per se* and plant species. There was also a trend toward higher bacterial diversity in rhizosphere soils compared to the initial soil substrate as indicated by the Shannon–Wiener and Simpson’s indices (**Supplementary Figure S1**). The observed increase in alpha diversity was paralleled by a trend toward larger bacterial and archaeal 16S rRNA gene copy numbers in the rhizosphere soils (Supplementary Table S1), as revealed by qPCR analysis across seven selected plant species representing the five analyzed plant orders.

**FIGURE 1:**
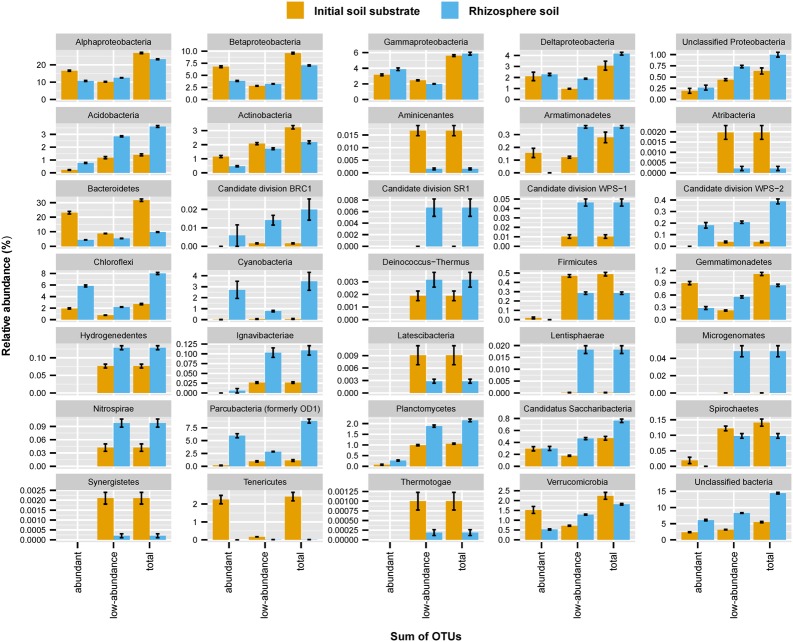
Mean relative abundance of bacterial phyla or proteobacterial classes across the initial soil substrate and rhizosphere soils as averaged across all analyzed plant species (*n* = 19). Error bars represent one standard deviation. The contribution of on average abundant (≥0.1%) and low-abundance (<0.1%) OTUs to changes between soils as well as their sum are displayed separately. A detailed distribution of bacterial phyla per plant species is given in **Supplementary Figure S2**.

The initial soil substrate and rhizosphere soils were dominated by bacteria belonging to the phyla Proteobacteria (classes Alpha-, Beta-, Gamma-, and Deltaproteobacteria), Bacteroidetes, Chloroflexi, Parcubacteria, Actinobacteria, Acidobacteria, Verrucomicrobia, Planctomycetes or unclassified bacteria, with each of these phyla constituting more than 1% relative abundance of the detected 16S rRNA genes (**Figure 1** and **Supplementary Figure S2**). Major relative abundance differences in the rhizosphere soils as compared to the initial soil substrate were evident in a massive decrease of Bacteroidetes (from 31.4 ± 3.6% to 9.7 ± 1.0%) and Tenericutes (from 2.4 ± 1.0% to 0.01 ± 0.00%), which was paralleled by an increase in Acidobacteria (from 1.4 ± 0.3% to 3.6 ± 0.3%), Chloroflexi (from 2.7 ± 0.5% to 8.0 ± 0.8%), Parcubacteria (from 1.1 ± 0.8% to 8.8 ± 1.7%), Planctomycetes (from 1.1 ± 0.1% to 2.2 ± 0.3%), and unclassified bacteria (from 5.5 ± 0.9% to 14.4 ± 1.1%). Interestingly, these increases in relative abundance were not solely driven by normalized sequence counts of abundant OTUs (≥0.1% relative abundance per OTU) but to a substantial part also by the sum of normalized sequence counts of low-abundance OTUs (<0.1% relative abundance per OTU). For example, the summed relative abundance of low-abundance OTUs within the Acidobacteria made up 1.2% (± 0.4%) and 2.9% (± 0.2%) of communities in the initial soil substrate and rhizosphere soils, respectively. In contrast, the summed relative abundance of abundant Acidobacteria OTUs made up only 0.2% (± 0.1%) and 0.8% (±0.2%) of communities in the initial soil substrate and rhizosphere soils, respectively. Among the 35 bacterial phyla and proteobacterial classes that were identified, 15 showed an increase in relative abundance that was dominated by the sum of normalized sequence counts of low-abundance OTUs (**Figure 1**). This included numerically dominant phyla such as the Acidobacteria and Planctomycetes as well as phyla that represented only a minority of the bacterial community such as Lenthisphaerae or Deinococcus-Thermus (**Figure 1**).

**FIGURE 2:**
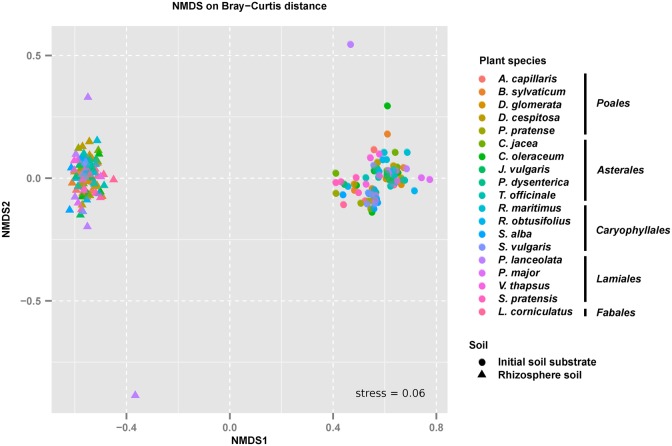
Beta-diversity of bacterial communities in the initial soil substrate and the corresponding rhizosphere soils of 19 different grassland plant species. Separation of bacterial communities in the analyzed soils is shown according to a non-metric multidimensional scaling (NMDS) analysis based on Bray–Curtis distances. Individual points represent sequenced soil replicates.

The large difference in bacterial beta diversity between the initial soil substrate and the rhizosphere soils was also evident when analyzed at the level of individual OTUs. Here, a clear separation of replicated initial soil substrate and rhizosphere soils could be observed in a NMDS plot irrespective of the analyzed plant species (**Figure 2**). This shift was highly significant as determined by a PERMANOVA analysis (**Table 2**). 55% of the variation in bacterial community composition among soil samples was explained by the incubation over 14 weeks. In addition, variation in bacterial community composition among plant species was also significant but much smaller (7%). A significant interaction between plant-species identity and incubation indicated that bacterial communities responded differently depending upon plant species, and this interaction explained 7% of the variation (**Table 2**). Since this result may reflect different starting communities among species and soils, we also tested for dispersion of communities among samples. Dispersion did not differ significantly among species (*F*_17,154_ = 0.494, *p* = 0.957). However, it did differ significantly between the initial soil substrate and rhizosphere soils (*F*_1,170_ = 10.607, *p* = 0.001), which reflects the less variable bacterial community composition in rhizosphere soils as compared to the initial soil substrate (**Figure 2**).

**Table 2:** Permutational analysis of variance (PERMANOVA) assessing dissimilarity of rhizosphere bacterial communities according to soil incubation and plant species identity (using a Bray–Curtis dissimilarity matrix).

	df	MS	*F*-value	*R*^2^
*Whole community*				
Soil incubation	1	19.671	251.56***	0.55
Species identity	17	0.155	1.99***	0.07
Soil incubation × species identity	17	0.1489	1.90***	0.07
Residual	136	0.078		
*Subset of specific responders (after incubation)*				
Species identity	17	0.187	1.60***	0.29
Residual	68	0.116		
*Subset of common responders (after incubation)*				
Species identity	17	0.084	1.73***	0.30
Residual	68	0.048		


*Significance of tests indicated by ^∗∗∗^*p* < 0.001; ns, not significant (*p* > 0.05). df, degrees of freedom, MS, mean squares, *F*-value, value compared to the *F* distribution, *R*^*2*^, proportion of variation explained.*

Interestingly, there was no significant correlation between phylogenetic distance among plant species and rhizosphere bacterial community dissimilarity (Mantel ρ = -0.09, *n* = 86, *p* = 0.99). This strongly suggests that bacterial communities were no more dissimilar between distantly related (spanning five different plant orders), than they were between more closely related plant species (e.g., of the same genus). Variation in bacterial community composition in the rhizosphere soils was also not significantly explained by the amount of biomass accumulated by plants during the incubation period of 14 weeks (*F*_1,84_ = 1.684, *p* = 0.082, *R*^2^ = 0.02).

### Positive Plant-specific Responders Constitute on Average 1% of the Bacterial OTU Richness in the Rhizosphere

**FIGURE 3:**
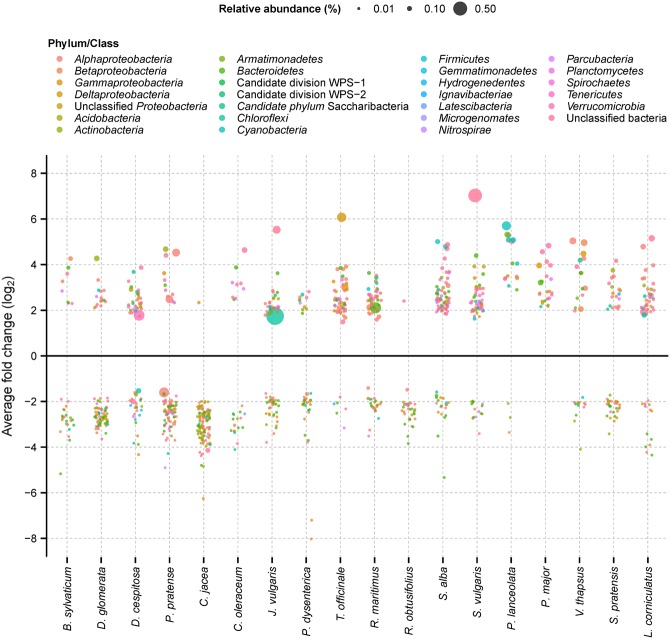
Bacterial OTUs (97% sequence identity) that responded significantly by relative abundance change according to one plant species only (plant-specific responders). Each point represents an OTU with the size of the points being proportional to the mean relative abundance of OTUs in the plant-specific rhizosphere soils. Point colors indicate the taxonomic affiliation of plant-specific responders. Further details are provided in Supplementary Table S2.

Each plant species had a small subset of 18–111 OTUs that responded specifically (either positive or negative) to that plant species only (**Figure 3**). The number of specific positive responders ranged from only 1 OTU up to 71 OTUs, which averaged 0.9% of observed OTUs per plant (range: 0.03–2.3%). On average, 91% of these positively responding OTUs were of low relative abundance (range: 60–100%). These low-abundance OTUs represented in 12 of 18 analyzed plant species also the first or second strongest positive responder in terms of log-fold change (e.g., unclassified Betaproteobacteria for *Brachypodium sylvaticum* and *Deschampsia cespitosa* or an unclassified Sinobacteraceae for *Centaurea jacea*) (Supplementary Table S2). This was also reflected in their summed relative abundance. Here, the sum of normalized sequence counts of positively responding low-abundance OTUs dominated numerically over positively responding abundant OTUs in 12 of the 18 analyzed plant species (Supplementary Table S2).

Plant-specific positive responders were mainly affiliated to the Alphaproteobacteria, unclassified bacteria, Bacteroidetes, Parcubacteria, Betaproteobacteria, or Verrucomicrobia (in descending order, ≥20 responders). However, there was no clear trend toward a certain phylogenetic lineage among the top or overall positive responding OTUs per plant species. For example, commonly observed members of the Rhizobiales (Alphaproteobacteria) were present only in 11 out of 18 plant species among the positive responding OTUs and members of the Flavobacterales (Bacteroidetes) in 10 plant species only (Supplementary Table S2).

The number of plant-species specific negative responders ranged from 0 to 110 OTUs per plant species (**Figure 3**), which averaged 1.0% of observed OTUs per plant (range: 0.0–3.6%). Again, their summed relative abundance was dominated by low-abundance OTUs in 13 of 18 analyzed plant species (Supplementary Table S2). The majority of these OTUs were affiliated to the Bacteroidetes, Alphaproteobacteria, unclassified bacteria, Betaproteobacteria, Gammproteobacteria, and Acidobacteria (in descending order, ≥20 responders). An overwhelmingly large fraction of these OTUs were of low relative abundance in the rhizosphere soils (average: 99%, range: 92–100%) (Supplementary Table S2).

These results were corroborated by a PERMANOVA analysis, showing a significant effect of plant-species identity on the observed plant-specific responders (**Table 2**). Interestingly, plant-species identity explained only 29% of total variation within plant-specific responders (both positive and negative). This indicates that while all these OTUs responded significantly to only one plant species, there was still a large amount of variation in abundance of these OTUs among soils of the other plant species. This also likely explains why there was no clear separation of the positive plant-specific responders according to plant phylogeny in a NMDS analysis (**Supplementary Figure S3**). This conclusion was further supported by a Mantel test on plant species-specific responses. Here, no significant correlation was observed between plant phylogeny and bacterial community dissimilarity of plant species-specific responders (Mantel ρ = -0.02, *n* = 86, *p* = 0.70).

### Common Responders Are Dominated by Chloroflexi, Parcubacteria, and Proteobacteria

A subset of 340 OTUs (9–13% of observed OTUs per soil replicate) differed significantly between the initial soil substrate and rhizosphere soils across all plant species. This included 127 and 213 OTUs that responded by increasing and decreasing in relative abundance, respectively (**Supplementary Figure S4**). Among the 21 phyla or proteobacterial classes that harbored common responders, 9 contained positive as well as negative responders with the Alphaproteobacteria and the Bacteroidetes being the most prominent ones. Phyla or classes that harbored exclusively positive responders were dominated by the Chloroflexi and Parcubacteria (out of seven phyla/classes in total), while exclusively negative responders were restricted to the Tenericutes, Spirochaetes, Hydrogenedetes, and unclassified Proteobacteria (**Supplementary Figure S4**).

In rhizosphere soils, the sum of normalized sequence counts of all common positive responders corresponded on average to a relative abundance of 26.1% of the total bacterial community (in comparison to 0.3% in the initial soil substrate). Roughly half (55%) of the positively responding OTUs were abundant, while the other half (45%) were of low-abundance (Supplementary Table S3). The most abundant positively responding OTUs belonged to the Chloroflexi (OTU4, 1.8% relative abundance), Parcubacteria (OTU7, 1.3% relative abundance), Cyanobacteria (OTU11, 1.0% relative abundance), and Gammaproteobacteria (OTU15, 1.0% relative abundance) (**Supplementary Figure S4** and Table S3). In contrast, normalized sequence counts of all common negative responders corresponded in sum to only 1.3% relative abundance of the total bacterial community in rhizosphere soils (in comparison to 44.4% in the initial soil substrate) and belonged exclusively to low-abundance OTUs. The largest relative abundance declines among negative responders were observed among members of the Bacteroidetes (OTUs 1, 8, 9, 10) and Tenericutes (OTU3), which were all present at 1% to 4% relative abundance in the initial soil substrate (Supplementary Table S3).

Interestingly, a significant amount (30%) of the variation in bacterial community composition of the common responders was explained by plant-species identity (**Table 2**). This suggests that even though these OTUs responded in the rhizosphere of all plants in a similar way, the magnitude of those responses differed according to plant species.

## Discussion

### Plant-specific Rhizosphere Bacteria belong Mainly to the Rare Biosphere

Plant-species identity is one of several factors that shapes the rhizosphere bacterial community (e.g., Miethling et al., 2000; Smalla et al., 2001; Garbeva et al., 2008; Berg and Smalla, 2009; Bezemer et al., 2010; Pendergast et al., 2013). For example, a comparison of plants belonging to different plant orders like oilseed rape (*Brassica napus*, order Brassicales), potato (*Solanum tuberosum*, order Solanales) and strawberry (*Fragaria ananassa*, order Rosales) revealed obvious differences in DGGE-fingerprint profiles of the bacterial community inhabiting the rhizosphere (Smalla et al., 2001; Costa et al., 2006). The same was true when comparing plants belonging to the same order-like rank like the herbeceous species hound’s tongue (*Cynoglossum officinale*) and spear thistle (*Cirsium vulgare*) (both *Asterids*) (Kowalchuk et al., 2002) or the same family like the grass species Indian ricegrass (Stipa hymenoides), James’ galleta (Hilaria jamestii), and drooping brome (Bromus tectorum) (all belonging to the *Poaceae*) (Kuske et al., 2002). The majority of such studies used fingerprinting techniques, which allowed differentiation of abundant bacteria at low phylogenetic resolution. In our study, we expanded investigation of the root bacteriome to the large bacterial diversity represented by low-abundance bacteria (Dohrmann et al., 2013; Nuccio et al., 2016; Saleem et al., 2016; Shi et al., 2016) and analyzed how this rare biosphere shapes the plant species-specific root bacteriome.

Already at the level of individual bacterial phyla (or proteobacterial classes), it was evident that changes in the rare biosphere drive a substantial part of the bacterial community shift between the initial soil substrate and rhizosphere soils. Approximately 40% of the detected bacterial phyla and proteobacterial classes showed an increase in relative abundance that was dominated by the sum of normalized sequence counts of low-abundance OTUs (**Figure 1**). This was equally true for phyla that represented a numerically small proportion of the bacterial population (e.g., the Lenthisphaerae) as for numerically abundant phyla (e.g., the Acidobacteria). Contrary to one of our hypotheses, the composition of established rhizosphere communities did not show a significant imprint of host plant phylogeny (see Mantel test results). Indeed, only a small subset of the rhizosphere bacterial community was plant-species specific. This included both abundant OTUs, as was already known from previous studies (e.g., Miethling et al., 2000; Smalla et al., 2001; Garbeva et al., 2008; Berg and Smalla, 2009; Bezemer et al., 2010; Pendergast et al., 2013), but to a large extent also low-abundance OTUs. The significant positive response of low-abundance OTUs to a specific host plant indicates population growth and metabolic activity, especially in the context of increasing overall bacterial and archaeal populations (**Figure 3** and Supplementary Table S1) Thus, our results contribute to a growing body of evidence that bacterial populations that belong to the rare biosphere are not just part of a dormant ‘seed’ bank that can be ‘activated’ upon environmental change but can actually directly contribute to ongoing processes in the soil and other environments (Pester et al., 2010; Großkopf et al., 2012; Lynch and Neufeld, 2015; Hausmann et al., 2016; Jousset et al., 2017). Especially in the highly structured root environment with secondary and primary roots including root hairs and caps, it can easily be envisaged that such overall low-abundance populations may constitute on a microscale locally abundant bacteria that could have an important effect on plant growth.

On average, roughly 1% of rhizosphere bacterial OTUs were promoted by the specific plant-host with an equally large part being suppressed (**Figure 3** and Supplementary Table S2). However, plant species explained only 29% of total variation within these plant-specific responders (**Table 2**). This is likely a result of most of these plant-specific responders sustaining individual population sizes of low relative abundance. While for their specific host plant the response was significant, these OTUs were likely at the detection limit of our sequencing effort for the other plants, with rather sporadic and inconsistent encounters that resulted in high variance among samples within species. Nonetheless, these results contribute to the evidence that plant species leave a discernible imprint on their respective rhizosphere soil.

In our study, rhizosphere soil was defined as soil attached to the roots. As such, we integrated over a gradient of soil that had a variable impact by plant roots (from strong to weak). Rhizosphere soil that is in close proximity to plant roots (a few millimeter) can be expected to harbor more plant-specific bacteria at potentially higher relative abundance. However, the small numbers of plant-specific responders in our study was comparable to studies that analyzed effects of different cultivars of the same plant species. Among different *A. thaliana* cultivars, 1.5% of observed bacterial OTUs in the endophyte-compartment were genotype-specific (Lundberg et al., 2012) and in *Solanum tuberosum* about 4% of rhizosphere- and root-inhabiting OTUs were genotype-dependent across two different analyzed soils (Weinert et al., 2011). Thus, the number of bacteria responding to specific plants does not appear to increase from genotypes of the same species (Weinert et al., 2011; Lundberg et al., 2012) through to species of different orders (this study). If these small subsets of responding bacteria influence plant performance, then these findings would agree with a recent meta-analysis, which found no plant phylogenetic signal in the strength of plant-soil feedback effects (Mehrabi and Tuck, 2015).

### The Rhizosphere Bacterial Community is Strongly Influenced by Common Responders

The observed community shifts between the initial soil substrate and rhizosphere soils (**Figure 2**) were strongly influenced by a roughly equally large number of abundant and low-abundance OTUs that significantly responded in the presence of all tested plant species (**Supplementary Figure S4** and Table S3). These common responders were represented by 340 OTUs, which constituted in terms of their summed relative abundance one quarter of the total bacterial community across the rhizosphere of all analyzed plant species. Due to our experimental design, we cannot differentiate between OTUs that responded to plant growth only as opposed to those that responded solely due to abiotic conditions, e.g., recolonization of provided substrate such as vermiculite or washed sand. However, since it is known that plants strongly influence their rhizosphere (Marschner et al., 1986; Dennis et al., 2010; Berendsen et al., 2012; Bulgarelli et al., 2013; van der Putten et al., 2013), at least part of these common responders likely responded to plant growth.

Among the common positive responders, the two most abundant OTUs (>1.3% relative abundance) belonged to the little-explored bacterial phyla Chloroflexi and Parcubacteria (formerly candidate phylum OD1). All 11 Chloroflexi OTUs among the common responders, including the most abundant common responder OTU, were affiliated to the Anaerolineaceae (subphylum 1) within the Chloroflexi. Cultivated representatives of the Anaerolineaceae have a heterotrophic lifestyle, being able to degrade carbohydrates and/or peptides and have been isolated from habitats like rice paddy soil, tundra meadow soil and anaerobic sludge (Costello and Schmidt, 2006; Yamada and Sekiguchi, 2009). The 11 Chloroflexi common responders of this study changed by 10–200 fold and constituted per OTU a relative abundance of 0.06–1.79% of the final rhizosphere bacterial community, with seven of these OTUs having relative abundancies of >0.1% (**Supplementary Figure S4**). As such, they may represent major generalists driving organic carbon mineralisation in the rhizosphere.

The second most abundant common responder OTU belonged to the Parcubacteria, with 17 additional Parcubacteria OTUs responding positively during the incubation irrespective of the host plant (8–200 fold changes; **Supplementary Figure S4**). All known members of this phylum possess very small and streamlined genomes lacking biosynthesis pathways for nucleotides, amino acids, fatty acids and co-factors like quinones and flavins. Therefore, a mutualistic or parasitic lifestyle has been proposed for these bacteria (Wrighton et al., 2012; Kantor et al., 2013; Rinke et al., 2013; Nelson and Stegen, 2015). This could also be the case in our study although we cannot exclude a direct association of these bacteria with plant roots. It is intriguing that 12 of these common Parcubacteria responders had a moderate or even high relative abundance (>0.1% relative abundance) with one of them being the second most abundant common responder (1.3% relative abundance, **Supplementary Figure S4**). Such large bacterial populations are generally postulated to be directly involved in major ecosystem functions like the carbon and energy flow (Pedrós-Alió, 2012; Lynch and Neufeld, 2015). Our results suggest that symbionts associated with microorganims can hitchhike this principle allowing them to build up large populations without being directly involved in the degradation of plant-derived material in the rhizosphere.

## Conclusion

Plants are known to modify the bacterial community in their close root vicinity (Berg and Smalla, 2009; Bulgarelli et al., 2013; Philippot et al., 2013). Our study shows that plant species identity is only a minor driver in this process with no significant imprint of plant phylogeny. The small number of rhizosphere bacterial OTUs that actually responded in a plant-species specific manner was dominated by members that sustain populations of low relative abundance. In addition, populations of low-abundance bacteria were in sum the major drivers of common responses at the phylum level. This adds to a growing body of evidence that the rare biosphere is not only a dormant seed bank but also harbors species that display enhanced metabolic activity, increase functional diversity, or increase community-wide species interactions (Jousset et al., 2017). Future studies need to demonstrate whether and how such low-abundance populations play an important role in affecting plant growth.

## Author Contributions

WD designed and performed experiments, analyzed the data and wrote the manuscript. MP designed experiments, analyzed the data and wrote the manuscript. JH performed experiments and contributed to writing the manuscript. MvK and ME contributed to designing experiments and to writing the manuscript.

## Conflict of Interest Statement

The authors declare that the research was conducted in the absence of any commercial or financial relationships that could be construed as a potential conflict of interest.

## Supplementary Material

**FIGURE S1** | Alpha diversity of analyzed samples when rarefied to an equal sampling depth of 16417 reads per replicate. Observed number of bacterial OTUs, Chao1 richness estimate, Shannon–Wiener and Simpson’s Index for the initial soil substrate and respective rhizosphere soils are displayed for each plant species.

**FIGURE S2** | Mean relative abundance of bacterial phyla, and in case of Proteobacteria classes, across the initial soil substrate and rhizosphere soils for each plant species.

**FIGURE S3** | Beta-diversity of bacteria in rhizosphere soils that responded positively to one plant species only. Separation of bacterial subcommunities in the analyzed soils is shown according to a non-metric multidimensional scaling (NMDS) analysis based on Bray–Curtis distances (stress = 0.19). Individual points represent sequenced soil replicates. *Agrostis capillaris* was excluded from statistical analysis due to an insufficient number of initial soil substrate replicates and is therefore not shown.

**FIGURE S4** | Bacterial OTUs (97% sequence identity) that significantly responded by relative abundance change between the initial soil substrate and respective rhizosphere soils across all plant species (common responders). OTUs are arranged by phyla, and in case of Proteobacteria by classes. Each point represents an OTU with the size of the points being proportional to the mean relative abundance of OTUs across all plant soil replicates in the rhizosphere soils.
