# Supplementary figures and images for: A Small Number of Low-abundance Bacteria Dominate Plant Species-specific Responses during Rhizosphere Colonization
Source: Front Microbiol. 2017 May 29;8:975. doi: 10.3389/fmicb.2017.00975 (PMC5447024; doi:10.3389/fmicb.2017.00975)

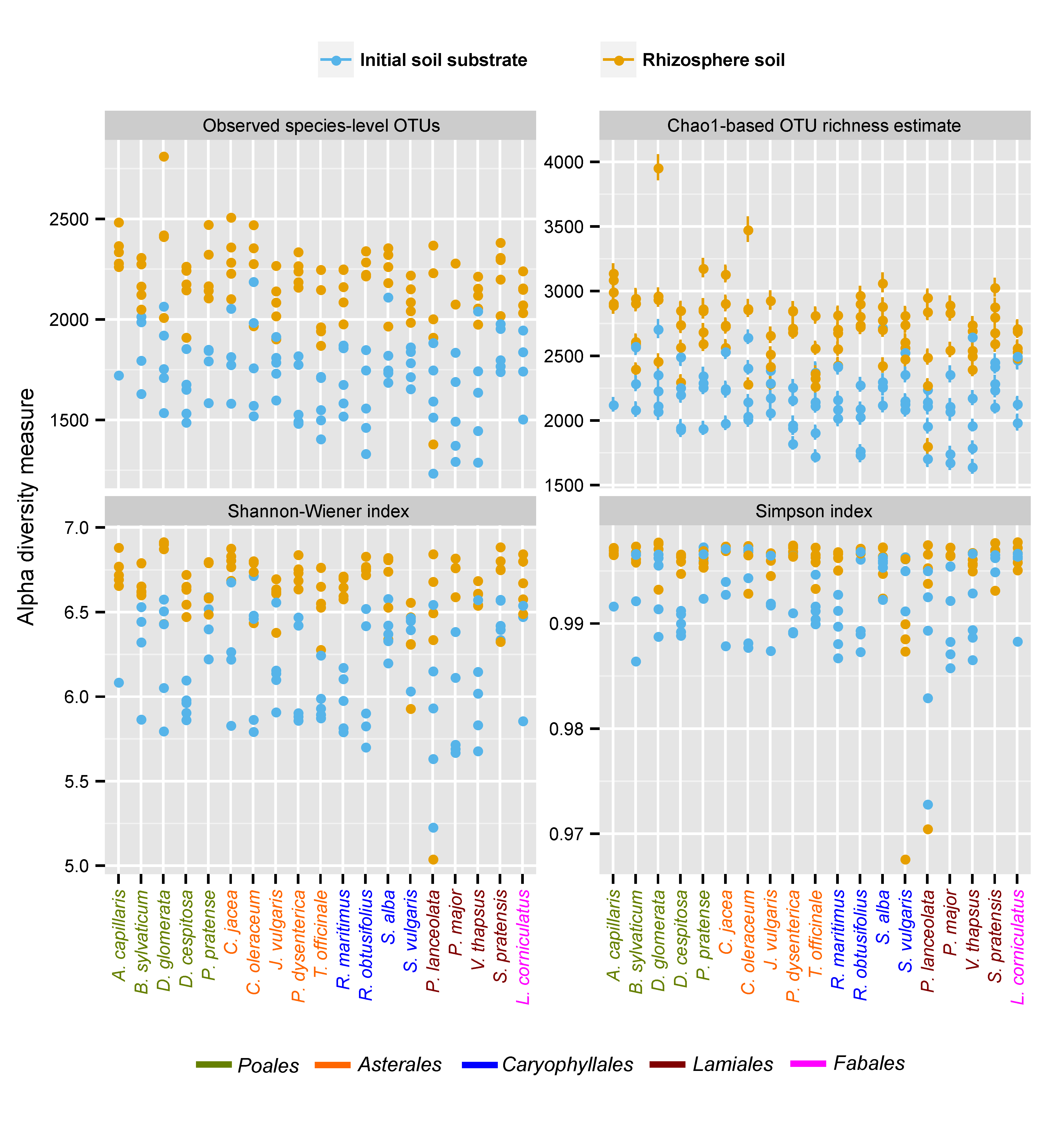

Supplement: Supplementary file 1 [file Image_1.TIF]

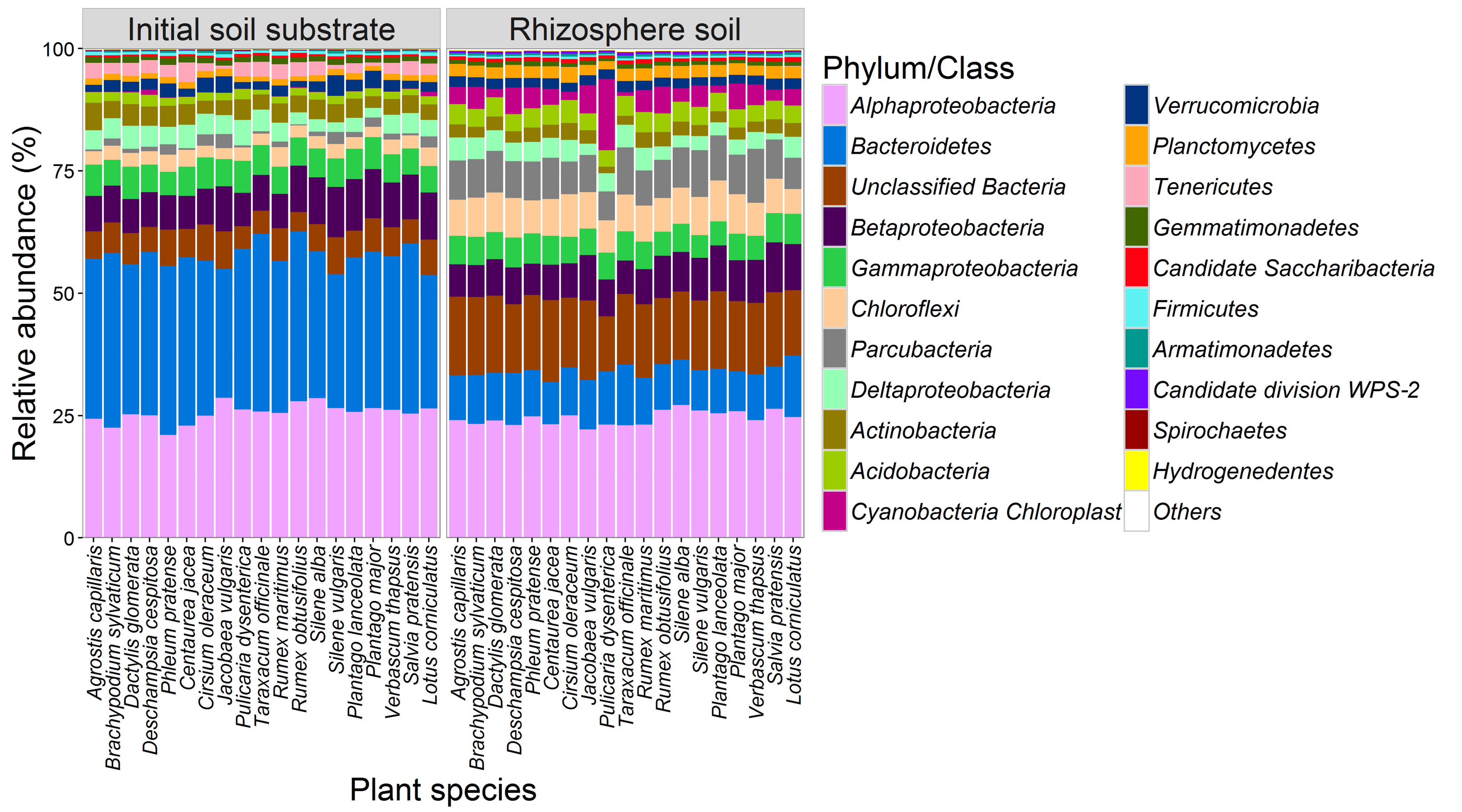

Supplement: Supplementary file 2 [file Image_2.TIF]

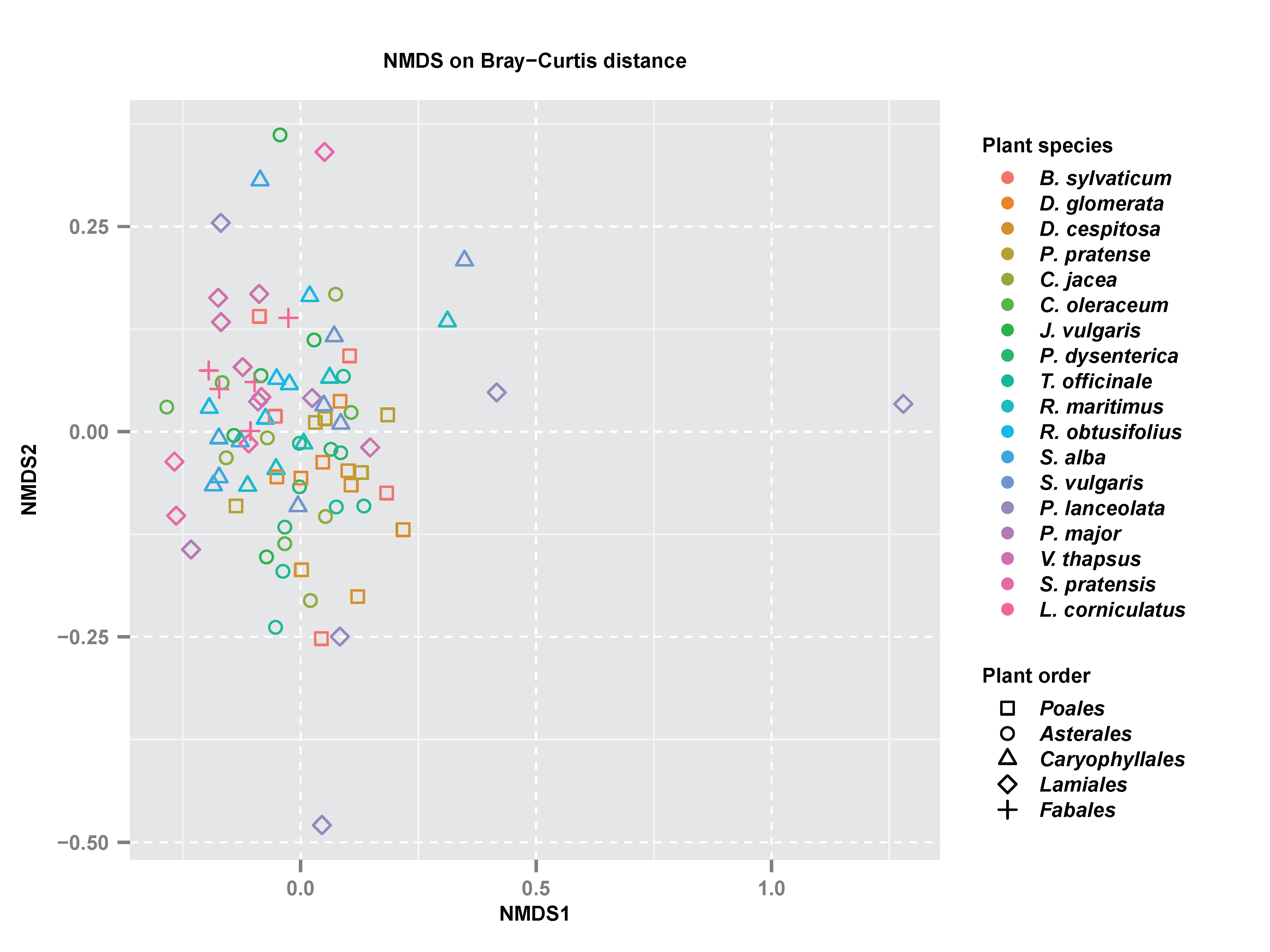

Supplement: Supplementary file 3 [file Image_3.TIF]

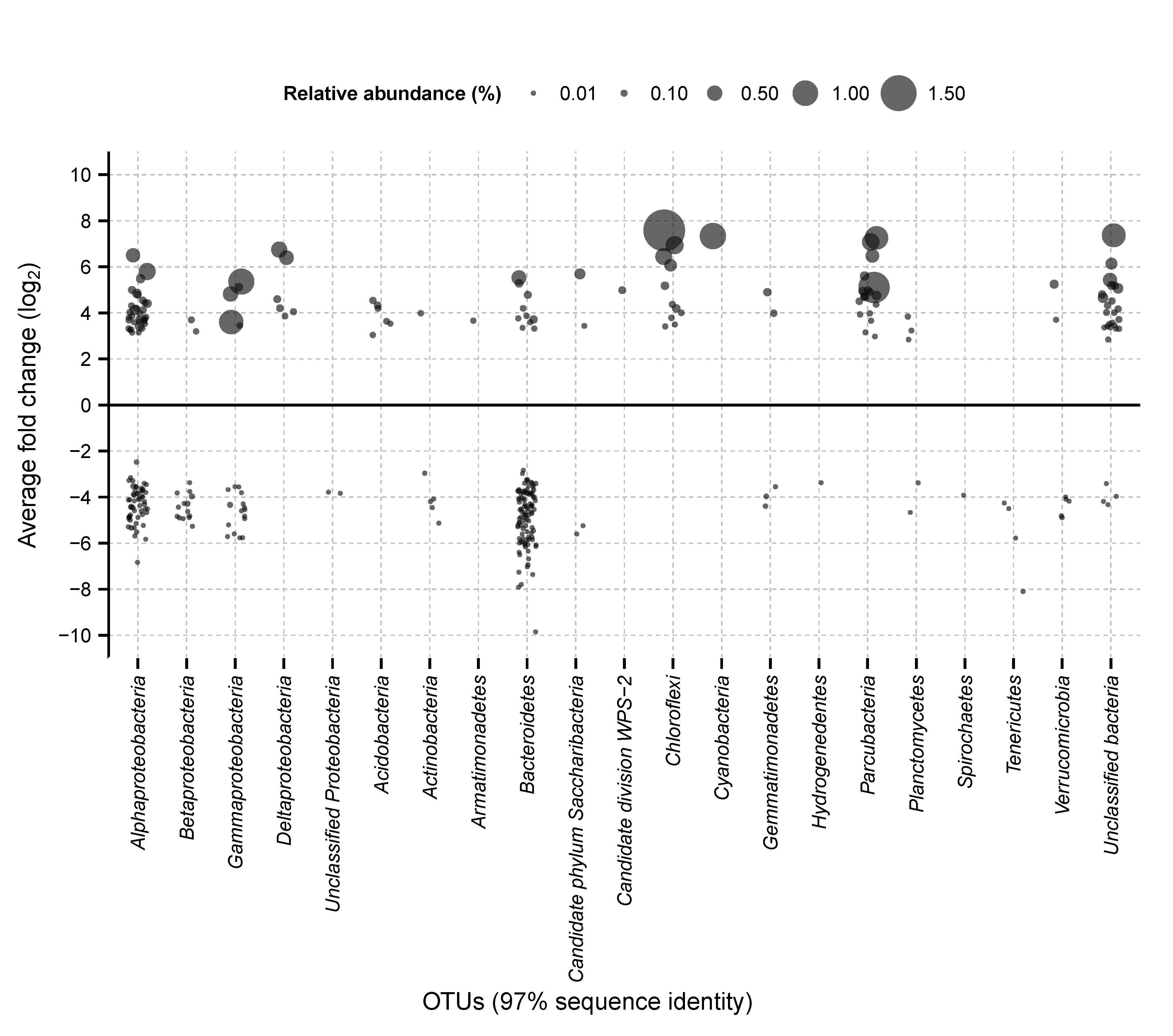

Supplement: Supplementary file 4 [file Image_4.TIF]
