# Supplementary material for: A Small Number of Low-abundance Bacteria Dominate Plant Species-specific Responses during Rhizosphere Colonization
Source: Front Microbiol. 2017 May 29;8:975. doi: 10.3389/fmicb.2017.00975 (PMC5447024; doi:10.3389/fmicb.2017.00975)
Supplement: Supplementary file 5 [file Table_1.PDF]

Table S1. Absolute abundance of most bacteria and archaea in the initial soil substrate and established rhizosphere soils for seven selected plant species as revealed by 16S rRNA gene-targeted qPCR. The number of analysed replicates per plant and time point ranged from 4 to 5 (see Table 1).

			**16S rRNA genes × 10^6 / g soil (wet weight)**	
**Species**	**Family**	**Order**	**inital**	**rhizosphere**
Phleum pratense	Poaceae	Poales	124.5 ± 44.2	251.9 ± 160.5
Centaurea jacea	Asteraceae	Asterales	98.7 ± 42.4	142.2 ± 67.3
Rumex obtusifolius	Polygonaceae	Caryophyllales	76.3 ± 31.2	238.5 ± 99.2
Silena alba	Caryophyllaceae	Caryophyllales	145.0 ± 48.3	184.3 ± 31.2
Plantago lanceolata	Plantaginaceae	Lamiales	87.1 ± 47.0	266.9 ± 134.3
Salvia pratensis	Lamiaceae	Lamiales	64.6 ± 25.3	249.1 ± 75.7
Lotus corniculatus	Fabaceae	Fabales	111.9 ± 58.5	206.2 ± 67.5

Table S2 (separate excel file)**.** Overview of OTUs that responded significantly to one plant species only (plant-specific responders) including taxonomic affiliation, log_2-fold change of relative abundance response and mean relative abundance in the initial soil substrate and respective rhizosphere soils.

Table S3 (separate excel file)**.** Overview of OTUs that responded significantly by relative abundance change between the initial soil substrate and respective rhizosphere soils across all plant species (common responders) including taxonomic affiliation, log_2-fold change of relative abundance response and mean relative abundance in the initial soil substrate and rhizosphere soils.
